# HNF4α-Mediated LINC02560 Promotes Papillary Thyroid Carcinoma Progression by Targeting the miR-505-5p/PDE4C Axis

**DOI:** 10.3390/biom15050630

**Published:** 2025-04-28

**Authors:** Yongcheng Su, Beibei Xu, Chunyi Gao, Wenbin Pei, Miaomiao Ma, Wenqing Zhang, Tianhui Hu, Fuxing Zhang, Shaoliang Zhang

**Affiliations:** 1 Department of Traditional Chinese Medicine and Xiamen Key Laboratory for Tumor Metastasis, Cancer Research Center, School of Medicine, Xiamen University, Xiamen 361102, China; 24520210157060@stu.xmu.edu.cn (Y.S.); 21620190154571@xmu.edu.cn (C.G.); 24520220157343@stu.xmu.edu.cn (W.P.); 24520221154758@stu.xmu.edu.cn (M.M.); wqzhang@xmu.edu.cn (W.Z.); thu@xmu.edu.cn (T.H.); 2 Department of Breast Surgery, The First Hospital of Xiamen University, School of Medicine, Xiamen University, Xiamen 361004, China; 3 Institute of Synthetic Biology, Shenzhen Institute of Advanced Technology, Chinese Academy of Sciences, Shenzhen 518055, China; bb.xu@siat.ac.cn; 4 Department of General Surgery, The First Hospital of Xiamen University, School of Medicine, Xiamen University, Xiamen 361004, China; 5 Shenzhen Research Institute of Xiamen University, Shenzhen 518057, China

**Keywords:** *LINC02560*, HNF4α, PTC, *miR-505-5p*, PDE4C, growth and metastasis

## Abstract

Papillary thyroid carcinoma (PTC) is the most common subtype of thyroid malignancy, and its progression is closely associated with patient outcomes. This study investigated the role of the long non-coding RNA *LINC02560* in the pathogenesis and aggressiveness of PTC through cell culture, transfection, RT-qPCR, Western blot analysis, and various functional assays, such as MTT, EdU, colony formation, wound healing, and Transwell migration assays. Our results revealed a significant upregulation of *LINC02560* in PTC tissues, correlating with poor prognosis in affected patients. Functional analyses demonstrated that silencing of *LINC02560* markedly inhibited the proliferation, migration, and invasion of the PTC cell lines, KTC-1, and BCPAP, whereas overexpression promoted these aggressive traits. Mechanistically, *LINC02560* acted as a competitive endogenous RNA, sponging *miR-505-5p* and alleviating its suppression on PDE4C degradation, thereby activating the P-AKT and epithelial–mesenchymal transition (EMT) signaling pathways. Additionally, HNF4α was identified as a transcription factor capable of enhancing the expression of *LINC02560*. In conclusion, our findings elucidate the critical HNF4α/*LINC02560*/*miR-505-5p*/PDE4C axis in PTC pathology, presenting this regulatory network as a promising biomarker combination and potential therapeutic target to improve patient outcomes and survival rates, warranting further clinical investigation to validate these insights and support the development of targeted therapies in PTC management.

## 1. Introduction

Among the various endocrine malignancies, thyroid cancer (TC) is the most common, with a steady increase in global incidence observed in recent decades [1]. Approximately 85% of TC cases are attributed to papillary thyroid carcinoma (PTC) [2]. Although PTC generally has a favorable prognosis, 20–30% of patients experience recurrence, and 5–10% develop progressive and refractory disease, potentially leading to life-threatening outcomes due to invasiveness and metastasis [3,4]. Advanced PTC has a 5-year survival rate of just 59% [5], with tumor invasiveness and metastasis significantly contributing to a poor prognosis. These challenges underscore the urgent need to explore the mechanisms underlying PTC invasiveness and to develop novel anticancer therapies to improve PTC progression [6].

Long non-coding RNAs (lncRNAs) are RNA sequences exceeding 200 nucleotides in length that do not produce proteins [7,8,9,10]. Several studies have shown that lncRNAs play essential roles in a range of physiological and pathological processes, including the onset and advancement of cancer [11,12,13,14], chemotherapy resistance [15], tumor energy metabolism regulation [16], and immune response modulation [17]. For example, the lncRNA lnc-TALC regulates temozolomide (TMZ) resistance by competitively binding to *miR-20b-3p* and promoting c-Met expression [15], whereas lung cancer progression can be promoted by the reprogramming of energy metabolism through the overexpression of lncRNA *IGFBP4-1* [14]. Despite a growing understanding of the functions of lncRNAs, their roles in PTC remain largely unknown [18].

In this study, we propose a novel signaling network in which *LINC02560*, induced by HNF4α, affects PTC tumorigenesis and metastasis via the *miR-505-5p*/PDE4C axis. The graphical abstract of this study is shown in the Graphical Abstract section.

## 2. Materials and Methods

### 2.1. PTC Sample Collection

Ten pairs of PTC and adjacent normal tissue samples were acquired from the First Affiliated Hospital of Xiamen University. Pathological examinations confirmed all samples as PTC. The Institutional Review Board of the First Affiliated Hospital of Xiamen University granted approval for this study, and informed consent was obtained from all patients.

### 2.2. Cell Lines, Cell Culture, and Transfection

The KTC-1 and BCPAP PTC cell lines were provided by the Chinese Academy of Sciences Cell Bank, located in Shanghai, China. Cells were cultured in 1640 medium, supplemented with 10% fetal bovine serum and maintained at 37 °C in an incubator with 5% CO2. Plasmids for overexpression and knockdown of HNF4α, as well as *LINC02560* and PDE4C, were constructed by Miaoling (Wuhan, China). A lentiviral vector system was used to establish cell lines with altered expression of *LINC02560*, HNF4α, and PDE4C. *miR-505-5p* mimics, and inhibitors, along with their corresponding negative controls, were obtained from Ribobio (Guangzhou, China). Transfection of these constructs and controls into KTC-1 and BCPAP cell lines was performed using the LabFect RNAi Transfection reagent (#T10110, LABLEAD, Beijing, China). Following transfection, the cells were left to incubate for 24 h prior to being harvested for further analyses. Detailed sequences of the constructs are listed in Table S1.

### 2.3. RT-qPCR and Western Blot Analysis

RT-qPCR and Western blot analyses were conducted as outlined in earlier descriptions [19,20]. YEASEN Biotech (Shanghai, China) supplied the RT-qPCR reagents used in these experiments. Table S2 contains the primer sequences used for RT-qPCR. The primary antibodies for E-cadherin, N-cadherin, Vimentin, AKT, P-AKT, and GAPDH were obtained from Proteintech (catalog numbers 20874-1-AP, 22018-1-AP, 10366-1-AP, 60203-2-Ig, 66444-1-Ig, and 5174, respectively). The dilution ratios were determined according to the manufacturer’s instructions.

### 2.4. MTT, EdU, and Colony Formation Assays

Cell proliferation was evaluated using the MTT assay, as previously described [19]. DNA synthesis in proliferating PTC cells was assessed using an EdU Imaging Kit (RiboBio, China), following the manufacturer’s protocol. Around 500 cells were placed in each well of the 6-well plates for the colony formation assay and incubated at 37 °C for a duration of two weeks. Following PBS washes, cells were fixed with 4% paraformaldehyde for 15 min and stained with crystal violet. Microscopic counting was performed, including only colonies that had at least 50 cells. Each assay was independently replicated three times.

### 2.5. Wound Healing and Transwell Assays

Wound healing experiments were performed following previously published protocols [20]. For the Transwell assays, post-transfection, cells were seeded at 5000 cells/well into pre-equilibrated 8 μm pore Transwells (3374, Corning, New York, NY, USA) with inserts coated with (for invasion) or without (for migration) Matrigel (#356237, BD Biosciences, San Jose, CA, USA). Following 24 h of incubation, non-migrating cells in the upper chamber were removed using a cotton swab, and cells that had migrated to or invaded the lower chamber were fixed in 4% paraformaldehyde for 10 min and stained with crystal violet. The number of migrated or invaded cells in six randomly selected fields was counted under a microscope.

### 2.6. RNA Immunoprecipitation (RIP) and Chromatin Isolation by RNA Purification (ChIRP)

The RIP assay was performed using a RIP Kit (Bersin Bio, New York, NY, USA). Antibody-conjugated beads (IgG/AGO2) were incubated with specified cells in RIP lysis buffer at 4 °C overnight. The complexes were thoroughly washed, extracted, purified, and analyzed using qRT-PCR. The ChIRP procedure was performed using a ChIRP Kit (Bersin Bio) according to the manufacturer’s protocol. The ChIRP probes are listed in Table S3.

### 2.7. Luciferase Reporter Assays

KTC-1 and BAPAP cells were cultured in 24-well plates. Various cell groups received co-transfections of *miR-505-5P* along with the corresponding NC mimics. After 48 h, the cells were lysed, and the activities of firefly and renilla luciferase were assessed. Firefly luciferase activity was used as a control to determine relative activity.

### 2.8. Immunohistochemistry (IHC)

To evaluate protein expression in the tissue samples, immunohistochemical staining was performed on formalin-fixed, paraffin-embedded sections. These sections, which were cut to 4 μm thick, underwent deparaffinization with xylene followed by rehydration through a graded ethanol series. To perform heat-induced epitope retrieval, the sections were placed in a citrate buffer at pH 6.0 and microwaved for 15 min. To quench the endogenous peroxidase activity, sections were treated with 3% hydrogen peroxide for 10 min. Slides were then exposed to a primary antibody specific to Ki-67 (1:400 dilution, CST, Danvers, MA, USA, #94490) overnight at 4 °C, diluted according to the manufacturer’s recommendations. After thorough washing with PBS for one hour at room temperature, the sections were treated with biotinylated secondary antibodies. Sections were then counterstained with hematoxylin, dehydrated, and mounted for microscopic examination (Leica Aperio Versa 200, Leica Camera AG, Somme, Germany).

### 2.9. Animal Experiments

All animal experiments were performed according to the protocols approved by the Animal Care and Use Committee of Xiamen University (Xiamen, China). Female nude mice (BALB/c, 15–20 g, 5–6 weeks old) were obtained from the SLAC Laboratory Animal Centre (Shanghai, China) and maintained under specific pathogen-free conditions at the Xiamen University Laboratory Animal Centre (Xiamen, China). For proliferation assays, BALB/c nude mice received subcutaneous injections of KTC-1 cells (5 × 10^6^). Weekly weighing of the mice was conducted, and they were euthanized five weeks after injection. Finally, the tumor volumes and weights were measured.

### 2.10. Bioinformatic Analysis

We downloaded the TCGA-THCA dataset (502 THCA tissue samples, including 58 normal adjacent samples), gene expression data, and corresponding clinical information from The Cancer Genome Atlas (TCGA, http://portal.gdc.cancer.gov, accessed on 22 June 2022). The lncLocator database (http://www.csbio.sjtu.edu.cn/bioinf/lncLocator, accessed on 8 January 2023) was used to identify the cell location of lncRNAs. Furthermore, the LncBase database (https://diana.e-ce.uth.gr/lncbasev3/home, accessed on 22 June 2022) and starBase database (https://rnasysu.com/encori/, accessed on 22 June 2022) were used to predict the lncRNA targets associated with miRNA. Binding sites between miRNAs and LncRNAs were predicted using the RNAhybrid database (https://bibiserv.cebitec.uni-bielefeld.de/rnahybrid/, accessed on 8 January 2023). The possible target transcription factors of lncRNAs were obtained with the use of RNAInter database (http://www.rnainter.org/IntaRNA/, accessed on 8 January 2023). Transcription factor binding sites were predicted using the JASPAR Transcription Factor Binding Site database (https://jaspar.genereg.net/, accessed on 8 January 2023).

### 2.11. Statistical Analyses

All statistical analyses were performed as appropriate. All experimental data conformed to a normal distribution. Estimates of variance for each group of data were not performed before statistical analyses were performed. The variance was similar among the control groups. Data are presented as the mean ± SEM of at least three independent experiments. Significance was calculated using two-tailed *t*-tests or one-way analysis of variance (ANOVA). Statistical significance was set at *p* < 0.05. Statistical analyses were performed using GraphPad Prism 8.0.

## 3. Results

### 3.1. LINC02560 Is Upregulated in PTC, Correlating with Malignant Progression and Poor Prognosis

**Figure 1:**
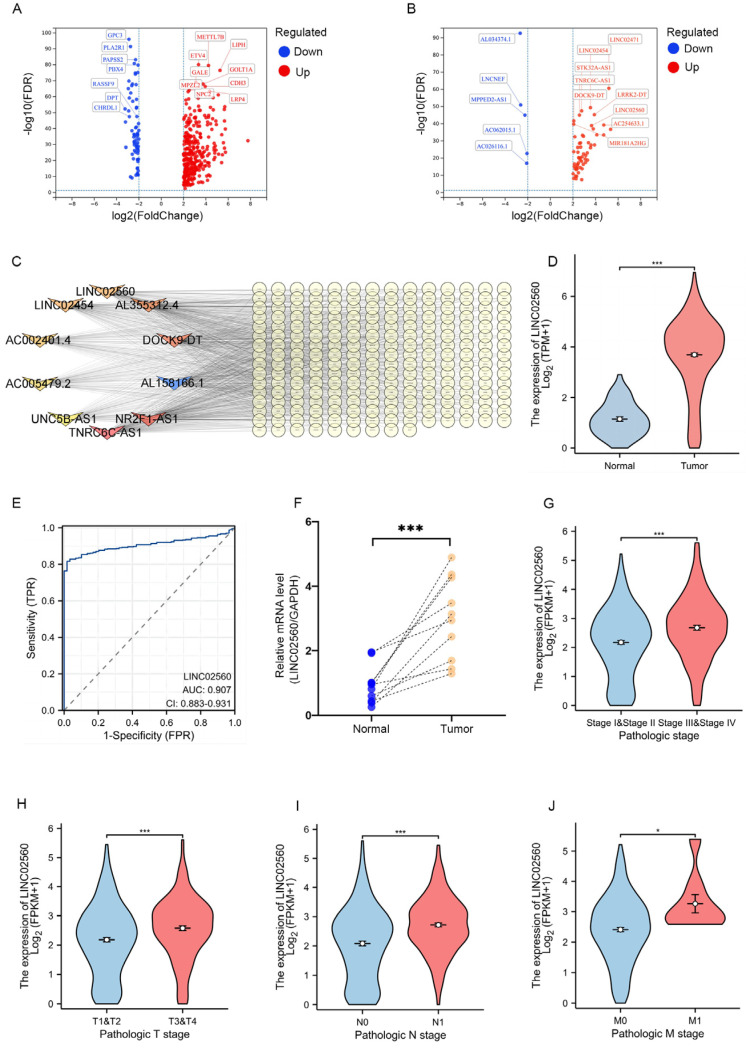
Higher *LINC02560* gene expression is associated with poor prognosis in thyroid cancer. (**A**,**B**) Volcano maps of differentially expressed (**A**) mRNAs and (**B**) lncRNAs in TCGA thyroid cancer tissues and adjacent normal tissues; red indicates gene upregulation, and blue indicates gene downregulation. (**C**) Thyroid cancer-related differentially expressed lncRNA-mRNA co-expression network signal network. (**D**) The expression of *LINC02560* was higher in thyroid cancer than in normal tissues based on TCGA database. (**E**) The ROC curves of *LINC02560* of thyroid cancer. (**F**) Relative expression of *LINC02560* in 10 paired PTC tissues and adjacent non-tumor tissues. (**G**–**J**) The expression levels of *LINC02560* were compared in various tumor stages: (**G**) TNM stage, (**H**) T stage, (**I**) N stage, (**J**) M grade. *** *p* < 0.001, * *p* < 0.05.

Using the TCGA database (TCGA-THCA), we conducted a comprehensive analysis of mRNA and lncRNA expression profiles in human PTC and adjacent non-cancerous tissues, identifying significantly differentially expressed genes (DEGs), as presented in Figure 1A,B. The volcano plots illustrated in these figures highlight the differential expression patterns of mRNAs and lncRNAs, respectively. We inferred the potential functions of the identified lncRNAs by constructing an lncRNA-mRNA co-expression network [21]. For co-expression network analysis, we selected differentially expressed mRNAs and lncRNAs based on a Pearson correlation coefficient threshold of 0.4, employing Cytoscape software (version 3.8.2) for network visualization. As presented in Figure 1C, 10 core lncRNAs were identified and integrated into a co-expression network, encompassing *AC002401.4*, *LINC02560*, *AL158166.1*, *NR2F1-AS1*, *UNC5B-AS1*, *AL355312.4*, *AC005479.2*, *DOCK9-DT*, *TNRC6C-AS1*, and *LINC02454*. Among the ten differentially expressed lncRNAs, the functions of seven have been previously reported in the context of thyroid cancer, with the exception of three lncRNAs: *AC002401.4*, *AL158166.1*, and *LINC02560*. Notably, among these three uncharacterized lncRNAs, *LINC02560* exhibited significant expression differences. Our analysis revealed that *LINC02560* was significantly upregulated in PTC, with its upregulation being the most pronounced among the three unreported lncRNAs. Therefore, we further assessed the differential expression of *LINC02560* between PTC tissues and adjacent normal tissues using the TCGA-THCA dataset. As illustrated in Figure 1D, *LINC02560* was significantly upregulated in PTC tissues, indicating its potential as a diagnostic biomarker, with an area under the ROC curve (AUC) of 0.907 (Figure 1E). This finding was corroborated by RT-PCR analyses, which demonstrated a notable increase in *LINC02560* expression across 10 pairs of PTC tissues (Figure 1F). Notably, the association of *LINC02560* expression with PTC progression was evidenced by increased levels in advanced stages (III or IV), compared to those in the early stages (I or II), concurrent with an escalation in expression correlating with advancing TNM stages (Figure 1G–J). Collectively, these observations indicate that increased *LINC02560* expression correlates with adverse prognosis in patients with PTC.

### 3.2. LINC02560 Promotes PTC Cell Proliferation, Movement, and Invasion

**Figure 2:**
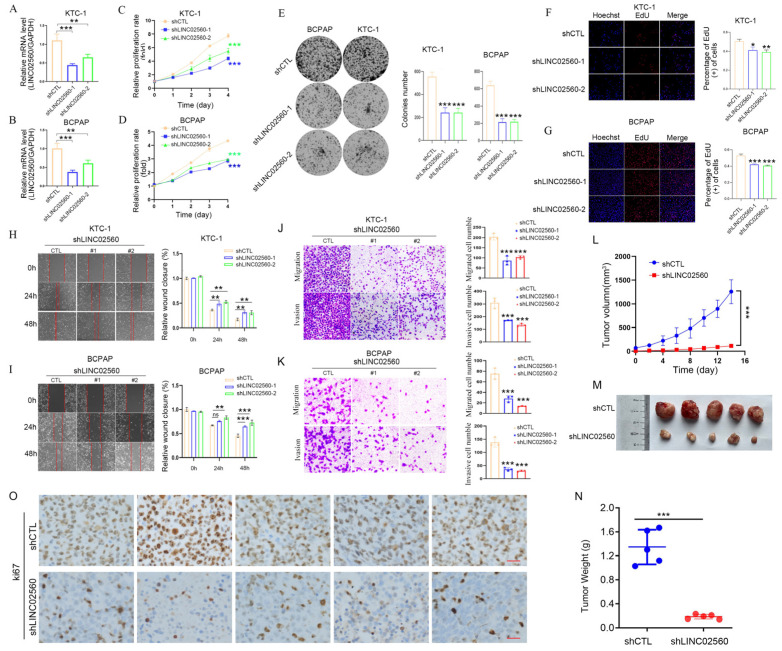
*LINC02560* knockdown inhibited the metastasis and growth of PTC cells in vitro. (**A**,**B**), qPCR assays verified the knockdown efficiency of *LINC02560* in PTC cells. (**C**,**D**). KTC-1 and BCPAP cell proliferation after knockdown of *LINC02560* by MTT assay. (**E**–**G**) Representative results of the colony formation (scale bar: 100 μm), and EdU assays (scale bar: 100 μm) in KTC1 and BCBAP cells after sh*LINC02560*-1 or sh*LINC02560*-2 transfection. (**H**–**K**) Representative images of PTC cell migration ability as shown by wound-healing assays ((**H**,**I**) scale bar: 100 μm) and migration assay ((**J**,**K**) scale bar: 100 μm). (**L**) Growth curves of sh-CTL and sh-*LINC02560* KTC-1 cells in nude mice after injection. (**M**,**N**) Images (**M**) and weights (**N**) of the tumors harvested from nude mice were shown. ((**O**) Scale bar, 20 μm) Ki-67 staining of tumors in sh-*LINC02560* group is downregulated compared with sh-CTL. * *p* < 0.05; ** *p* < 0.01; *** *p* < 0.001, ns. not significant.

To elucidate the functional implications of *LINC02560* in PTC progression, we infected TC cells (K1 and BCPAP) with lentiviral vectors designed for *LINC02560* knockdown, along with the corresponding control vectors (vector, shNC). Following infection, cells were subjected to a series of assays to evaluate their proliferation, migration, and invasion capabilities. Stable *LINC02560* knockdown (sh-*LINC02560*) cell lines were successfully established in KTC-1 and BCPAP cells through lentiviral transfection, as confirmed by the data presented in Figure 2A,B. The knockdown of *LINC02560* resulted in a significant reduction in cell viability, as assessed by the MTT assay, diminished colony formation capacity, and decreased EDU incorporation in both KTC-1 and BCPAP cells, as shown in Figure 2C–G. Moreover, wound healing (Figure 2H,I) and Transwell assays demonstrated a pronounced decrease in the migration and invasion abilities of cells with *LINC02560* knockdown, as shown in Figure 2J,K.

To expand our investigation to in vivo models, subcutaneous injections of KTC-1 cells, stably transfected with sh-*LINC02560* lentivirus or a control lentivirus, were administered to nude mice. Mice receiving *LINC02560* knockdown cells exhibited a significant reduction in tumor volume and weight compared to their control counterparts (Figure 2L–N). IHC analysis of the excised tumors further validated these findings, revealing a marked decrease in KI67 protein expression in the *LINC02560* knockdown group (Figure 2O).

*LINC02560* overexpression yielded results that were diametrically opposite to those observed following its knockdown. We engineered lentiviral vectors for *LINC02560* overexpression and corresponding controls to infect KTC-1 and BCPAP cells (Figure S1A,B). Subsequent analyses revealed that the overexpression of *LINC02560* led to a significant enhancement in cell viability in KTC-1 and BCPAP cells on the MTT assay, as well as increased colony formation capacity and augmented EDU incorporation (Figure S1C–G). Wound healing (Figure S1H,I) and Transwell assays further revealed a significant enhancement in the migration and invasion capabilities of cells that overexpressed *LINC02560*, as presented in Figure S1J,K. Similarly, when expanding our investigation to in vivo models, we found that mice injected with cells that overexpress *LINC02560* exhibited a notable rise in tumor volume and weight (Figure S1L–N), along with higher KI67 protein levels in the removed tumor tissues (Figure S1O). In summary, these in vivo findings corroborate the in vitro results, highlighting the pivotal role of *LINC02560* in PTC proliferation and metastasis.

Collectively, these data indicate that *LINC02560* significantly contributes to the advancement, migration, and invasion of PTC cells.

### 3.3. LINC02560 Influences PTC Cell Growth, Migration, and Invasion via EMT/AKT Pathway

**Figure 3:**
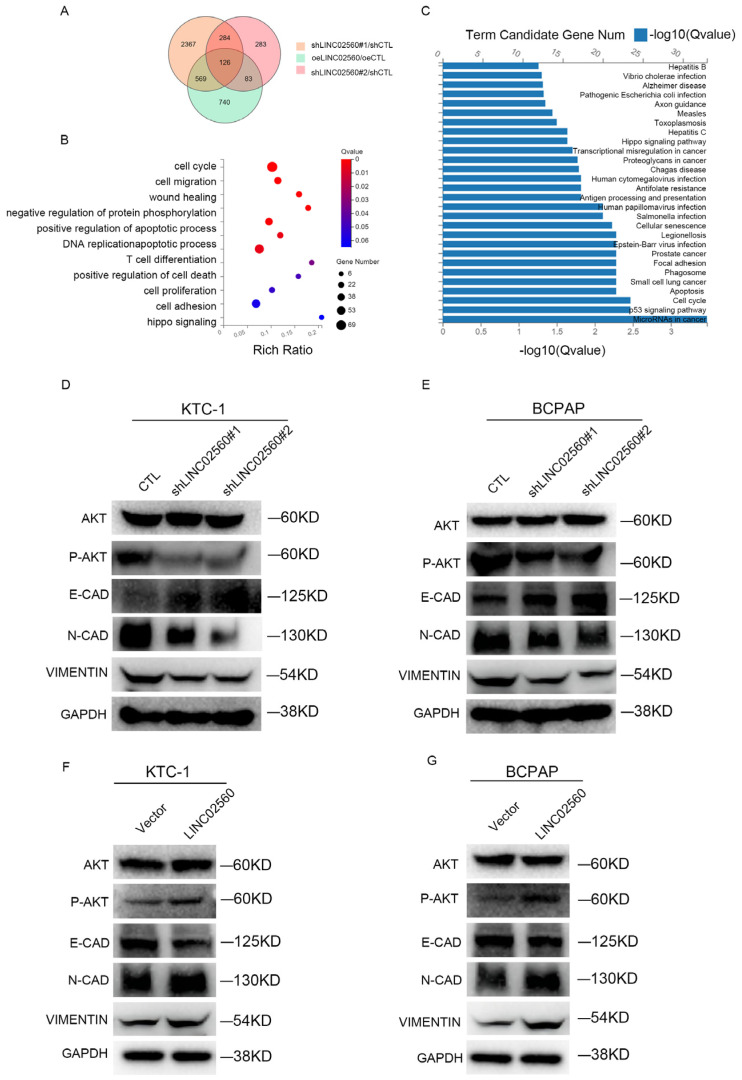
*LINC02560* promoted the metastasis and growth of PTC cells through the EMT via the AKT signaling pathway. (**A**) A Venn diagram shows the changes in differential genes (DEGs) before and after *LINC02560* knockdown or overexpression in KTC-1 cells; (**B**) GO enrichment analysis of DEGs in KTC-1 cells before and after *LINC02560* knockdown or overexpression; (**C**) KEGG enrichment analysis of DEGs in KTC-1 cells before and after *LINC02560* knockdown or overexpression. (**D**,**E**) Western blot analysis showing variations in proteins associated with the epithelial–mesenchymal transition (EMT) pathway (N-cadherin, Vimentin, E-cadherin) and the AKT pathway (phosphorylated AKT, P-AKT) in KTC-1 and BCPAP cells post-*LINC02560* knockdown. (**F**,**G**) Western blot analysis revealing changes in EMT pathway-related proteins (N-cadherin, Vimentin, E-cadherin) and AKT pathway components (P-AKT) in KTC-1 and BCPAP cells following *LINC02560* overexpression. Original images can be found in Supplementary Materials.

To elucidate the molecular mechanisms by which *LINC02560* modulates PTC progression, we performed RNA sequencing of KTC-1 cells after modulating *LINC02560* expression, either through overexpression or knockdown (Figure 3A). Subsequent Gene Ontology (GO) and Kyoto Encyclopedia of Genes and Genomes (KEGG) enrichment analyses of the DEGs underscored their involvement in cell proliferation, invasion, and metastasis (Figure 3B) and further highlighted their association with biological processes pivotal to oncogenesis, such as miRNAs in cancer, apoptosis, cell cycle regulation, the p53 signaling pathway, and other cancer-related pathways (Figure 3C). This aligns with the phenotypic manifestations associated with the alterations in *LINC02560* expression. Western blot analyses further corroborated these findings, revealing that *LINC02560* silencing attenuated the protein levels of N-cadherin and Vimentin, which are mesenchymal markers, while augmenting the expression of E-cadherin, an epithelial hallmark (Figure 3D,E). Moreover, the phosphorylation status of AKT, a key player in the AKT signaling pathway, was found to be positively proportional to *LINC02560* expression, decreasing upon knockdown and increasing upon overexpression (Figure 3F,G). These observations indicate that *LINC02560* may exert regulatory effects on PTC progression by modulating EMT-related genes and the AKT pathway.

### 3.4. LINC02560 Serves as a Molecular Sponge for miR-505-5P

**Figure 4:**
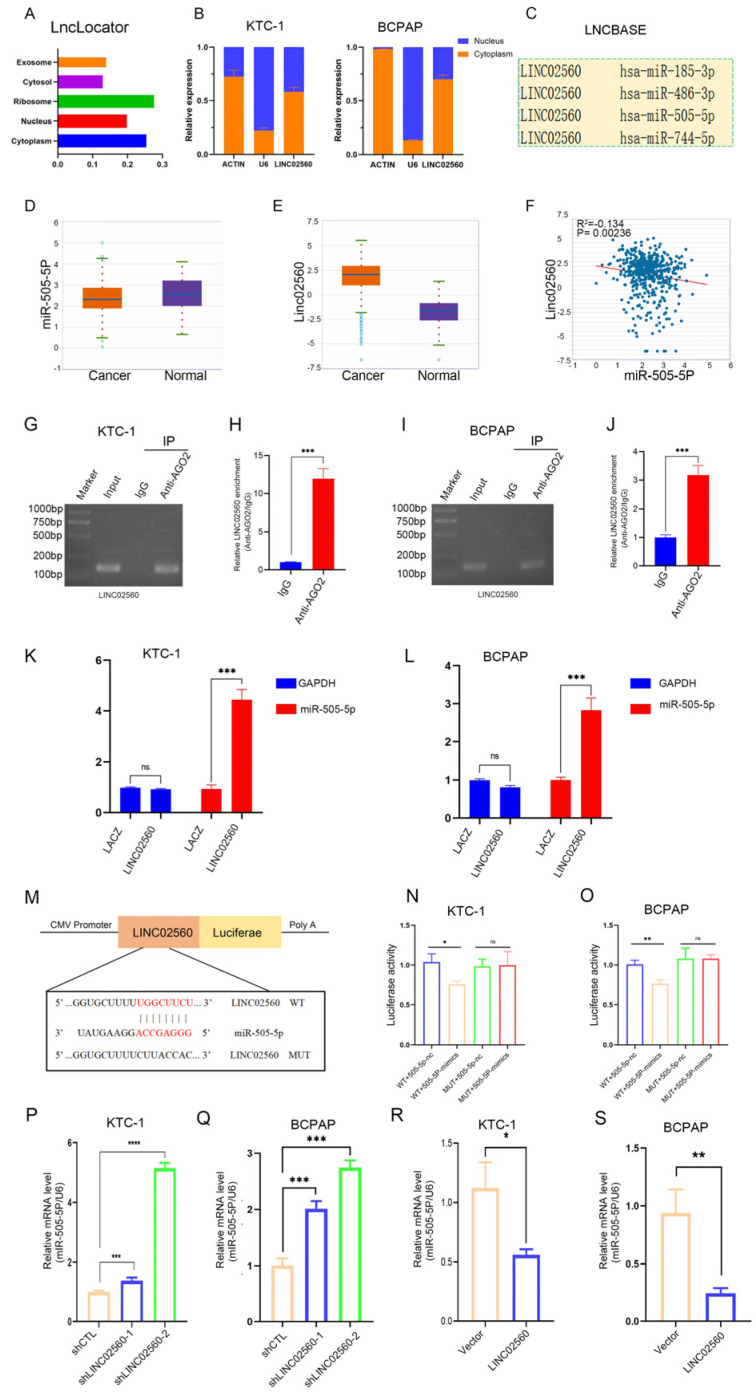
*LINC02560* acts as a competing endogenous RNA (ceRNA) and competitively absorbs *miR-505-5p*. (**A**) *LINC02560* was predicted to be located mainly in the cytosol using the bioinformatics tools in LncLocator. (**B**) qPCR analysis of *LINC02560* expression in the nucleus and cytoplasm of KTC-1 and BCPAP cells. β-actin, and U6 were used as endogenous controls. (**C**) LncBase database was utilized to predict the potential miRNAs that bind to *LINC02560*. (**D**,**E**) Relative expression of *miR-505-5p* and *LINC02560* in PC tissues and healthy tissues determined using the starBase database. (**F**) Relationship between *miR-505-5p* and *LINC02560* expression levels in PC samples determined using the starBase database. (**G**–**J**) RIP experiments were performed, and RT-qPCR assays and agarose gel electrophoresis were used to detect the enrichment of *LINC02560* to AGO2 in KTC-1 and BCPAP cells. (**K**,**L**) ChIRP assay analysis of the interaction between *LINC02560* and *miR-505-5P* in KTC-1 and BCPAP cells. (**M**) Binding sites between *miR-505-5p* and *LINC02560* were predicted using the RNAhybrid database. (**N**,**O**) Luciferase activity of the reporter plasmid containing either WT or MUT *LINC02560* 3′UTR in PTC cells following co-transfection with *miR-505-5p* mimic or NC mimic. (**P**,**Q**) q-PCR was performed to detect miR-505-5P expression after *LINC02560* knockdown in PTC cells. (**R**,**S**) q-PCR was performed to detect *miR-505-5P* expression after *LINC02560* overexpression in PTC cells. *, *p* < 0.05; **, *p* < 0.01; ***, *p* < 0.001, ****, *p* < 0.0001, ns. not significant.

To ascertain the subcellular localization of *LINC02560*, we employed lncLocator and conducted nuclear–cytoplasmic separation experiments, which revealed that *LINC02560* was primarily localized in the cytoplasm (Figure 4A,B). LncRNAs have been identified to function as the sponges of miRNAs so that the bound mRNAs are released to exert their functions. This mechanism is defined as the competing endogenous RNA (ceRNA) network. Given the cytoplasmic localization and stability of *LINC02560*, coupled with the known role of lncRNAs as miRNA sponges, we hypothesized that *LINC02560* functions as a miRNA sponge. We identified the following four candidate miRNAs that could interact with *LINC02560* through in silico analysis using the LNCBASE database: *miR-505-5p*, *miR-185-3p*, *miR-486-3p*, and *miR-744-5p* (Figure 4C). Furthermore, the starBase online database indicated that *miR-505-5p* was expressed at higher levels in normal tissues than in tumor tissues (Figure 4D), whereas *LINC02560* exhibited elevated expression in PTC tissues (Figure 4E), while the expression levels of *miR-505-5p* and *LINC02560* showed an inverse correlation (Figure 4F). We further predicted that *LINC02560* interacts with *miR-505-5p*, potentially regulating its activity in PTC.

To test this hypothesis, we performed RIP assays using Ago2 antibodies and control IgG antibodies. RIP assays demonstrated higher enrichment of *LINC02560* in the Ago2 group compared to the IgG group (Figure 4G–J), indicating that *LINC02560* interacts with miRNAs via the Ago2 protein. We further conducted CHIRP experiments on PTC cells using a biotin-labeled *LINC02560* probe, which showed a significant increase in the enrichment of *miR-505-5p* in the biotin-labeled *LINC02560* probe group relative to the control probe (Figure 4K,L). Using the online database RNAhybrid, we subsequently forecasted the binding sequence of *LINC02560* and *miR-505-5p*. We then constructed dual-luciferase reporter plasmids containing wild-type and mutant *LINC02560* sequences. In the wild-type plasmid, *miR-505-5p* mimics lowered luciferase activity, whereas the mutant plasmid remained unaffected (Figure 4M–O). Moreover, *LINC02560* knockdown increased *miR-505-5p* expression (Figure 4P,Q), whereas *LINC02560* overexpression decreased *miR-505-5p* expression (Figure 4R,S).

Collectively, these results confirm that *LINC02560* acts as a molecular sponge and directly binds to *miR-505-5p*.

### 3.5. miR-505-5P Partially Reverses the Tumorigenic Effect of LINC02560

**Figure 5:**
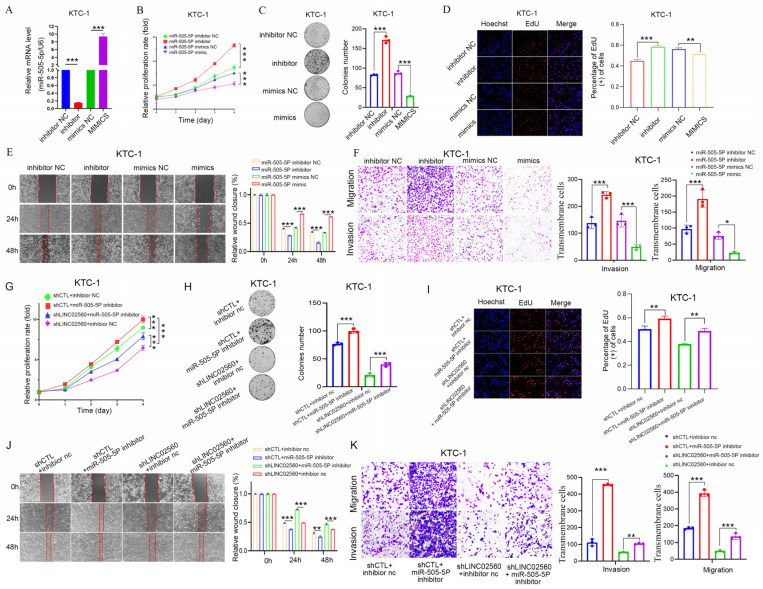
The tumor-inhibition effects of sh*LINC02560* could be reversed by *miR-505-5P* inhibitor. (**A**) Relative *miR-505-5p* levels in KTC-1 cell transfected with *miR-505-5p* mimics or inhibitor and their respective control. (**B**) KTC-1 cell proliferation after transfected with *miR-505-5p* mimics or inhibitor and their respective control by MTT assay. (**C**,**D**) Representative images of colony formation ((**C**) scale bar: 100 μm) and the EdU assays ((**D**) scale bar: 100 μm) in *miR-505-5p* mimics or inhibitor and their respective control transfected KTC-1 cell. (**E**,**F**) Representative images of KTC-1 cell migration ability as shown by wound-healing assays ((**E**) scale bar: 100 μm) and migration assay after transfected with *miR-505-5p* mimics or inhibitor and their respective control ((**F**) scale bar: 100 μm). (**G**–**J**) MTT (**G**), colony formation ((**H**) scale bar: 100 μm), and EdU assays ((**I**) scale bar: 100 μm) were performed to assess cell proliferation ability of each group. (**J**) The effect of sh*LINC02560* and *miR-505-5p* inhibitor on migration was examined by wound healing assays, scale bar: 100 μm. ((**K**) scale bar: 100 μm) The capacity of cell migration and invasion of each group was determined by Transwell assays. * *p* < 0.05; ** *p* < 0.01; *** *p* < 0.001.

To investigate the role of *miR-505-5p* in PTC, *miR-505-5p* mimics, inhibitors and controls were transfected into the KTC-1/BCPAP cell lines. The efficacy of the transfection was confirmed by measuring *miR-505-5p* expression levels 48 h post-transfection (Figure 5A and Figure S2A). MTT, colony formation, and EdU proliferation assays were used next to evaluate how *miR-505-5p* affects cell growth. The data revealed that the *miR-505-5p* mimics markedly inhibited the proliferation of KTC-1/BCPAP cells (Figure 5B–D and Figure S2B–D). Wound healing and Transwell migration assays further substantiated the inhibitory role of *miR-505-5p* mimics on cell migration and invasion, showing a significant suppression in KTC-1/BCPAP cells, whereas the application of *miR-505-5p* inhibitors exhibited antagonistic effects, thereby enhancing cellular proliferation, migration, and invasion (Figure 5E,F and Figure S2E,F). Collectively, these findings indicate that *miR-505-5p* serves as a crucial inhibitor of the growth, migration, and invasion of KTC-1/BCPAP cells.

To further investigate the interaction between *miR-505-5p* and *LINC02560*, rescue experiments were performed using *miR-505-5p* inhibitors and *LINC02560* knockdown vectors in KTC-1 cells. The results of EdU, MTT assays, and colony formation assays suggested that the suppression of cell growth caused by *LINC02560* knockdown were mitigated by the introduction of *miR-505-5p* inhibitors (Figure 5G–I). Moreover, analysis of the cellular capacity for invasion and migration revealed that the suppression caused by *LINC02560* knockdown was partially alleviated by treatment with *miR-505-5p* inhibitors (Figure 5J,K). These experimental results highlight the tumor-suppressive role of *miR-505-5p*, which effectively counterbalances the oncogenic potential of *LINC02560*.

In summary, the present evidence underscores the mechanism through which *LINC02560* aids in the malignant development of PTC cells by partially trapping *miR-505-5p*, thereby acting as a molecular sponge.

### 3.6. LINC02560 Positively Regulates PDE4C Expression in PTC Cells by Sponging miR-505-5p

**Figure 6:**
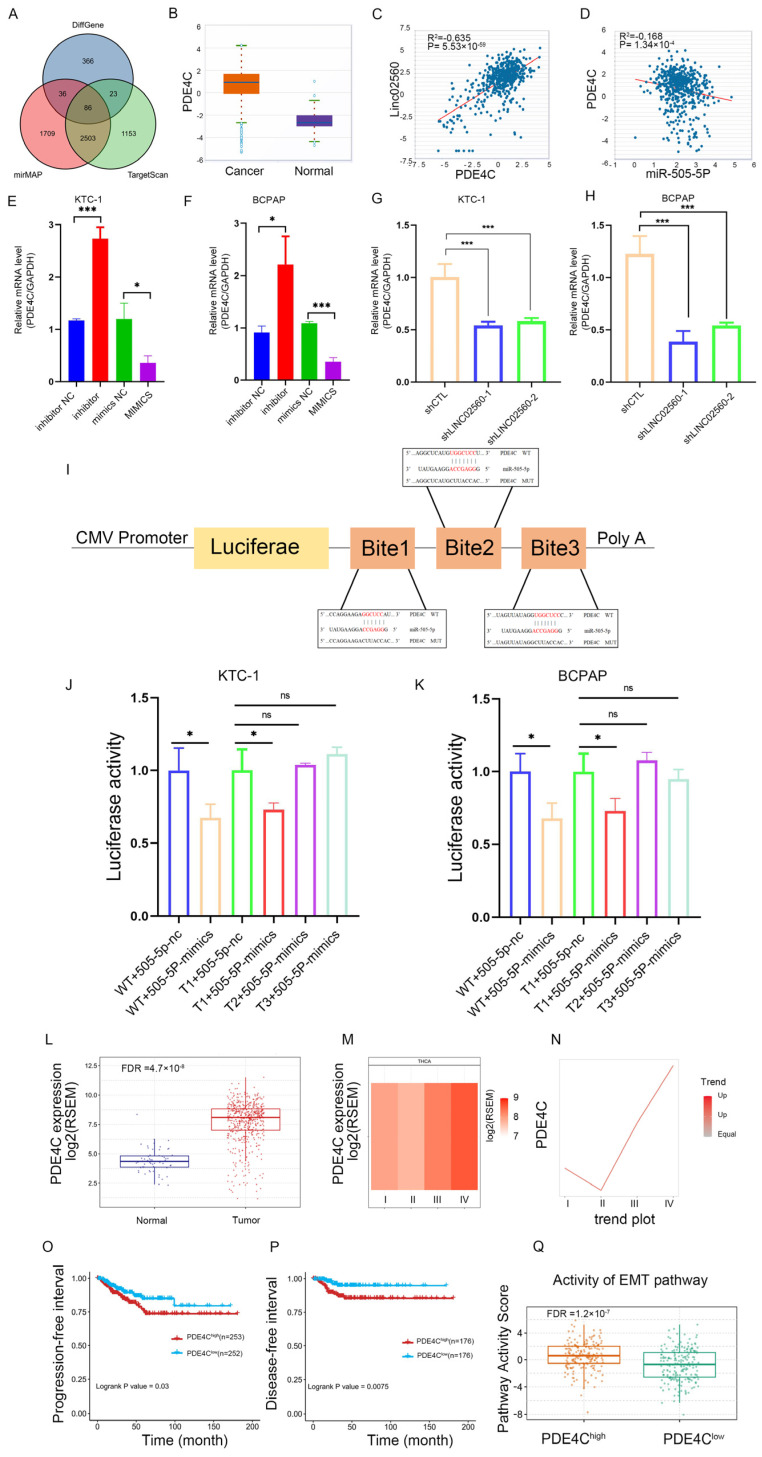
PDE4C is a direct target of *miR-505-5P*. (**A**) Venn diagram representing the potential targeted mRNAs of *miR-505-5P* by miMAP, TargetScan, and TCGA-THCA differentially expressed mRNAs. (**B**) Relative expression of PDE4C in thyroid cancer tissues and healthy tissues determined using the starBase database. (**C**) Relationship between PDE4C and *LINC02560* expression levels in thyroid cancer samples determined using the starBase database. (**D**) Relationship between PDE4C and *miR-505-5P*. expression levels in thyroid cancer samples determined using the starBase database. (**E**,**F**) RT-qPCR analyzed the relative expression of PDE4C in KTC-1 and BCPAP after transfecting with *miR-505-5P* mimic/mimic NC or *miR-505-5P* inhibitor/inhibitor NC. (**G**,**H**) RT-qPCR analyzed the relative expression of PDE4C in KTC-1 and BCPAP cells after transfecting with sh*LINC02560*/shCTL. (**I**–**K**) The luciferase activities in KTC-1 and BCPAP cells co-transfected with *miR-505-5p* mimic or mimic NC and luciferase reporters containing PDE4C 3′UTR WT or PDE4C 3′UTR MUT. (**L**) The expression of PDE4C was higher than in normal tissues in THCA based on GSCA database. (**M**,**N**) Heatmap (**M**) and trend plot (**N**) present the PDE4C expression among stages in THCA based on GSCA database. (**O**,**P**) Kaplan–Meier PFS and DFS curves of PDE4C in THCA using GSCA database. (**Q**) GSVA analysis showed the activity of EMT signaling pathway between high and low PDE4C expression groups in THCA based on GSCA database. *, *p*  <  0.05, ***, *p*  <  0.001, ns, not significant.

miRNAs can regulate downstream target genes by binding to their 3′-UTRs and causing degradation. To clarify the *LINC02560*/*miR-505-5p* axis and explore its downstream regulatory mechanism, we used the mirMAP (https://mirmap.ezlab.org/, accessed on 22 June 2022) and TargetScan (https://www.targetscan.org/, accessed on 22 June 2022) databases to predict the downstream target genes of *miR-505-5p*, identifying overlapping genes with differential mRNA expression in TCGA-THCA (Figure 6A). PDE4C was identified as a potential downstream gene of *miR-505-5p*. PDE4C was highly expressed in THCA tumor tissues (Figure 6B), and correlation analysis revealed a positive correlation between PDE4C and *LINC02560* (Figure 6C) and a negative correlation between PDE4C and *miR-505-5p* (Figure 6D). Using qRT-PCR, we subsequently demonstrated that transfection with *miR-505-5p* mimics or inhibitors in KTC-1 and BCPAP cells resulted in decreased PDE4C expression by *miR-505-5p* mimics and increased expression of the *miR-505-5p* inhibitor (Figure 6E,F). We subsequently examined PDE4C expression after *LINC02560* knockdown in KTC-1 and BCPAP cells and found that *LINC02560* knockdown reduced PDE4C expression (Figure 6G,H). We then used the TargetScan database to predict three potential binding locations for PDE4C and *miR-505-5p*. Dual-luciferase reporter plasmids were subsequently constructed with wild-type and mutant (T1, T2, and T3) forms of the PDE4C 3′UTR (Figure 6I). A significant reduction in luciferase activity was observed in the wild-type plasmid upon transfection with *miR-505-5p* mimics and T1-PDE4C reporter plasmids, whereas the mutant plasmids T2 and T3-PDE4C remained unaffected. Therefore, we speculate that *miR-505-5p* binds to sites 2 and 3 in the 3′UTR of PDE4C (Figure 6J,K). To further clarify the potential role of PDE4C in THCA occurrence and development, we analyzed the expression trend of PDE4C mRNA in TCGA-THCA based on the GSCA database, finding significant differences in THCA tumor tissues (Figure 6L). Furthermore, we observed a significant correlation between the expression of PDE4C and pathological stage, as demonstrated in Figure 6M,N. The heatmap and trend plot summarized the trend of PDE4C mRNA expression from the early stage to the late stage, while the pathological stage showed higher PDE4C mRNA expression in THCA. Survival curves also indicated that high PDE4C expression correlated with worse progression-free survival (PDS) (Figure 6O) and disease-free survival (DFS) (Figure 6P) in THCA, based on the GSCA database. We then performed Gene Set Enrichment Analysis (GSVA) on the low and high PDE4C expression groups in THCA. Remarkably, significant enrichment in EMT signaling pathways was observed in the group with elevated PDE4C expression (Figure 6Q). This conclusion is consistent with our previous findings that *LINC02560* influences PTC cell growth, migration, and invasion via the EMT pathway.

### 3.7. PDE4C Overexpression Rescues the Inhibitory Effects of LINC02560 Silencing on PTC Cell Proliferation, Migration, and Invasion

**Figure 7:**
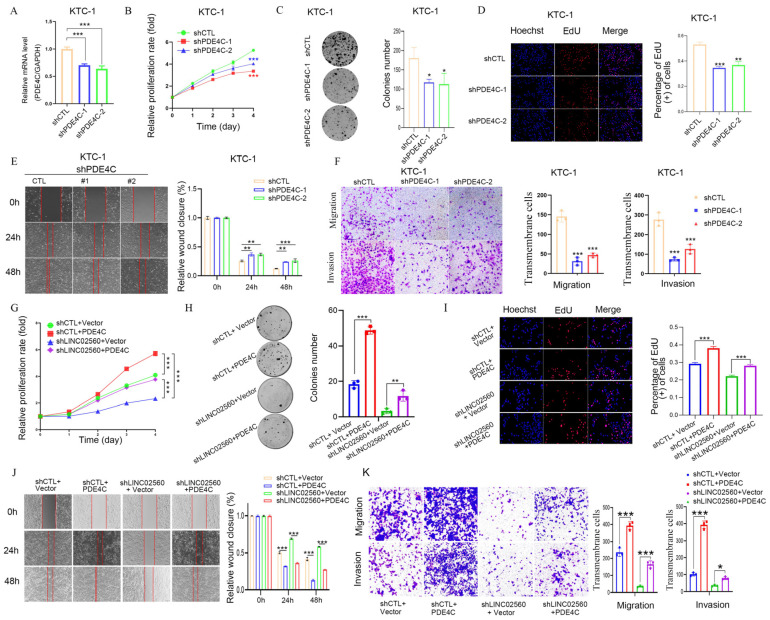
The tumor-inhibition effects of sh*LINC02560* could be reversed by PDE4C. (**A**) Relative PDE4C levels in KTC-1 cell transfected with PDE4C-sh1 or PDE4C-sh2. (**B**) KTC-1 cell proliferation after knockdown of PDE4C by MTT assay. (**C**,**D**) Representative images of colony formation ((**C**) scale bar: 100 μm) and the EdU assays ((**D**) scale bar: 100 μm) after PDE4C-sh1 or PDE4C-sh2 transfection KTC-1 cell. (**E**,**F**) Representative images of KTC-1 cell migration ability as shown by wound-healing assays ((**E**) scale bar: 100 μm) and migration assay ((**F**) scale bar:100 μm) after transfected with PDE4C-sh1 or PDE4C-sh2. (**G**–**J**) MTT (**G**), colony formation ((**H**) scale bar: 100 μm), and EdU assays ((**I**) scale bar: 100 μm) were performed to assess cell proliferation ability of each group. (**J**) The effect of sh*LINC02560* and PDE4C on migration was examined by wound healing assays, scale bar: 100 μm. ((**K**) scale bar: 100 μm) The capacity of cell migration and invasion of each group was determined by transwell assays. * *p* < 0.05; ** *p* < 0.01; *** *p* < 0.001.

To delineate the functional attributes of PDE4C within the context of PTC, we further utilized lentiviral vectors to induce PDE4C knockdown in KTC-1 and BCPAP cell lines, which resulted in the establishment of stable PDE4C-deficient cell lines (Figure 7A and Figure S3A). Subsequent assessments using MTT assays, colony formation experiments, and EdU incorporation assays revealed a substantial suppression of cellular proliferation in both KTC-1 and BCPAP cells following PDE4C knockdown (Figure 7B–D and Figure S3B–D). Furthermore, cellular motility was evaluated using wound healing assays (Figure 7E and Figure S3E), while the assessment of invasive capacity using Transwell assays (Figure 7F and Figure S3F) underscored the significant decrease in the migratory and invasive capabilities of cells lacking PDE4C, thereby implicating PDE4C as a pro-oncogenic entity in PTC.

Intrigued by the potential interplay between PDE4C and *LINC02560*, we subsequently investigated the alterations in PDE4C RNA expression following the modulation of *LINC02560* levels. To determine whether *LINC02560* exerts its effects by modulating PDE4C expression, we engineered a PDE4C overexpression vector and performed rescue experiments in the presence of *LINC02560* knockdown vectors. The suppressive impact of sh-*LINC02560* on PTC cell growth, movement, and invasion was reduced by PDE4C overexpression, as evidenced by the results of colony formation, EdU incorporation, and MTT assays, which collectively demonstrated that PDE4C overexpression counteracted the growth-inhibitory effects of *LINC02560* knockdown in PTC cells (Figure 7G–I). Moreover, with respect to the migratory and invasive capacities of PTC cells, PDE4C overexpression partially counterbalanced the inhibitory effect exerted by *LINC02560* knockdown (Figure 7J,K).

These findings collectively underscore a pivotal regulatory axis involving *LINC02560* and PDE4C and offer intriguing insights into the molecular dynamics involved in the pathophysiology of PTC.

### 3.8. HNF4α Increases LINC02560 Expression

**Figure 8:**
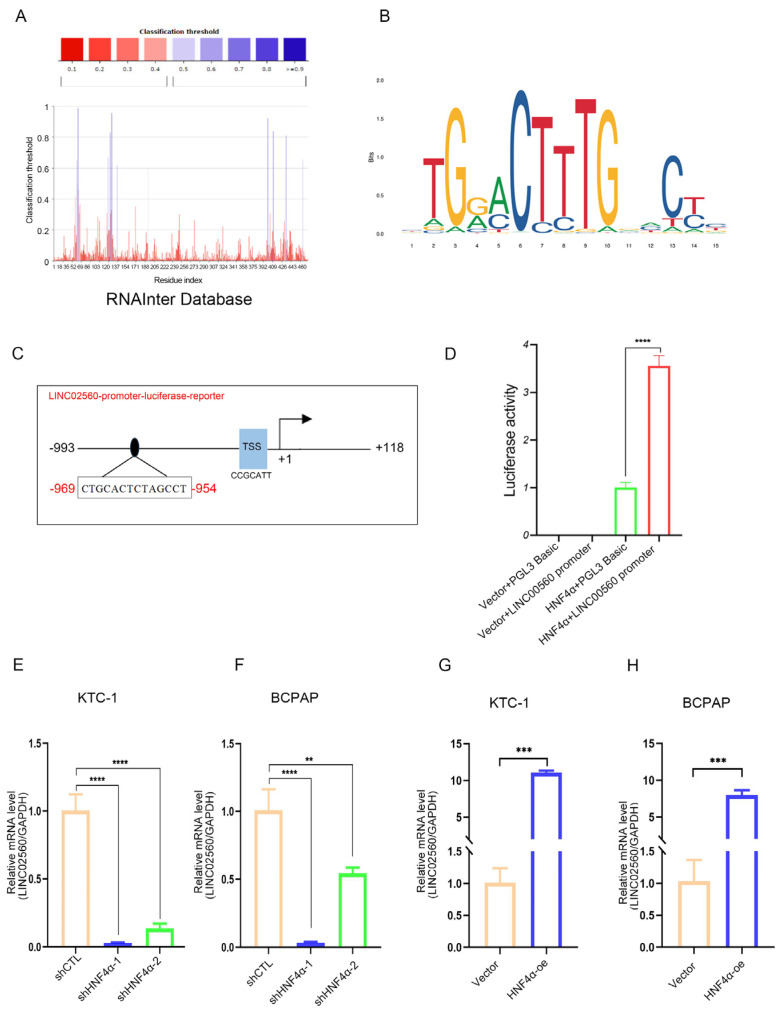
HNF4α regulates the expression of *LINC02560*. (**A**) Prediction of transcription factor HNF4α targeting *LINC02560* by the RNAInter Database. (**B**) Binding motif of HNF4α (from JASPAR). (**C**) Schematic depiction of the *LINC02560* promoter. A potential HNF4α binding site was located on the *LINC02560* promoter region. (**D**) Dual-luciferase reporter analysis of *LINC02560* promoter activity in 293T cells transfected with or without HNF4α. (**E**,**F**) q-PCR was performed to detect *LINC02560* expression after HNF4α knockdown in PTC cells. (**G**,**H**) q-PCR was performed to detect *LINC02560* expression after HNF4α overexpression in PTC cells. **, *p* < 0.01; ***, *p* < 0.001, ****, *p* < 0.0001.

To unravel the regulatory mechanisms governing *LINC02560* expression, the RNAInter online database was employed to discover transcription factors that might interact with *LINC02560*. Our analysis further indicated that HNF4α could potentially bind to *LINC02560* (Figure 8A). Subsequently, we employed the NCBI database (http://genome.ucsc.edu/, accessed on 8 January 2023) to search for potential sequences within the *LINC02560* promoter region and the JASPAR database to identify the motif sequence of the transcription factor HNF4α (Figure 8B). Through the JASPAR database, we located potential HNF4α binding sites on the promoter of *LINC02560*, leading us to hypothesize that HNF4α might play a role in the transcriptional regulation of *LINC02560*.

To substantiate this hypothesis, we engineered overexpression vectors for HNF4α¸ along with corresponding negative control vectors. Leveraging the identified binding sequence of HNF4α to the *LINC02560* promoter, we constructed luciferase reporter plasmids harboring the *LINC02560* promoter sequence (Figure 8C). Our findings demonstrated that HNF4α augmented the luciferase activity of the *LINC02560* promoter plasmid (Figure 8D). Conclusive RT-PCR validation further revealed that *LINC02560* expression was diminished following transfection with HNF4α knockdown plasmids (Figure 8E,F), whereas transfection with HNF4α overexpression plasmids resulted in elevated *LINC02560* expression in PTC cells (Figure 8G,H).

Collectively, these results indicate that HNF4α binds to the *LINC02560* promoter region, thereby exerting a regulatory effect on *LINC02560* expression.

### 3.9. HNF4α Induces LINC02560 Expression to Promote PTC Cell Growth, Migration, and Invasion

**Figure 9:**
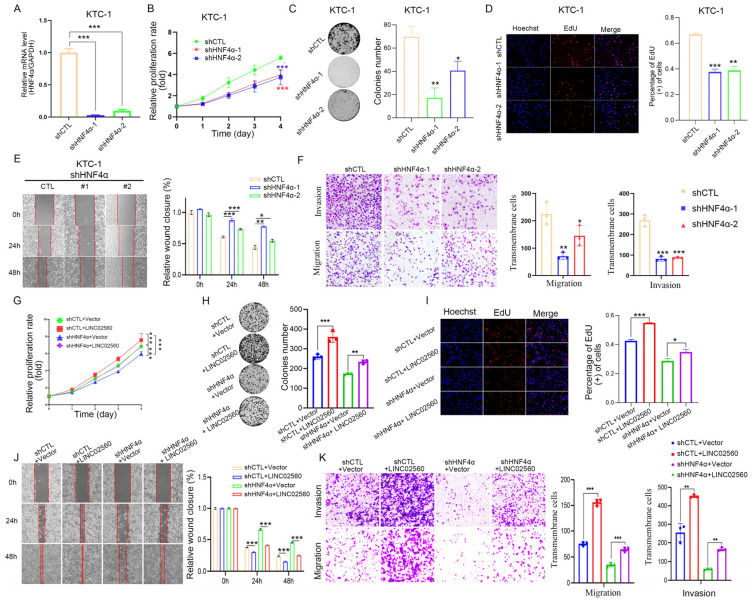
The tumor-inhibition effects of shHNF4α could be reversed by *LINC02560*. (**A**) Relative HNF4α levels in KTC-1 cell transfected with HNF4α-sh1 or HNF4α-sh2. (**B**) KTC-1 cell proliferation after knockdown of HNF4α by MTT assay. (**C**,**D**) Representative images of colony formation ((**C**) scale bar: 100 μm) and the EdU assays ((**D**), scale bar: 100 μm) after HNF4α-sh1 or HNF4α-sh2 transfection KTC-1 cell. (**E**,**F**) Representative images of KTC-1 cell migration ability as shown by wound-healing assays ((**E**) scale bar: 100 μm) and migration assay ((**F**), scale bar: 100 μm) after transfected with HNF4α-sh1 or HNF4α-sh2. (**G**–**J**) MTT (**G**), colony formation ((**H**) scale bar: 100 μm), and EdU assays ((**I**) scale bar: 100 μm) were performed to assess cell proliferation ability of each group. (**J**) The effect of shHNF4α and *LINC02560* on migration was examined by wound healing assays, scale bar: 100 μm. ((**K**) scale bar: 100 μm) The capacity of cell migration and invasion of each group was determined by Transwell assays. * *p* < 0.05; ** *p* < 0.01; *** *p* < 0.001.

To delve deeper into the functional implications of HNF4α in PTC, we employed lentiviral vectors to knockdown HNF4α in K1 and BCPAP cells, establishing stable HNF4α knockdown cell lines (sh-HNF4α) through lentiviral transfection (Figure 9A and Figure S4A). Subsequent MTT, colony formation, and EdU assays revealed significant suppression of cell viability, colony formation ability, and proliferative capacity in KTC-1 and BCPAP cells upon HNF4α knockout (Figure 9B–D and Figure S4B–D). Scratch assays (Figure 9E and Figure S4E) showed that cells with HNF4α knockdown exhibited a substantial decrease in migration and invasion, as shown in the Transwell assays (Figure 9F and Figure S4F), suggesting that HNF4α possesses oncogenic characteristics in PTC cells.

To ascertain whether the HNF4α oncogenic effects in PTC are mediated by *LINC02560*, we conducted rescue experiments by co-transfecting *LINC02560* overexpression or knockdown vectors with HNF4α overexpression or knockdown vectors. Colony formation, EdU, and MTT assays indicated that *LINC02560* overexpression effectively mitigated the inhibitory effects of HNF4α knockdown on PTC cell growth (Figure 9G–I). Regarding HNF4α’s impact on PTC cell invasion and migration, *LINC02560* overexpression partially restored the migratory and invasive capacities of HNF4α-knockdown cells (Figure 9J,K). In contrast, *LINC02560* knockdown attenuated the proliferative effects of HNF4α overexpression on PTC cell growth (Figure S5A–D) and abrogated the enhancement of PTC cell invasion and migration induced by HNF4α overexpression (Figure S5E–I). These experimental findings underscore that HNF4α functions as an oncogene in PTC, with its oncogenic activity being facilitated by the upregulation of *LINC02560* expression.

## 4. Discussion

PTC represents the most widespread type of thyroid malignancy [22]. Although generally bearing a favorable prognosis, specific variants such as the hypercellular subtype display more aggressive behavior, characterized by increased recurrence rates and poor prognostic outcomes [23]. In contemporary cancer research, lncRNAs have emerged as a focal point for research, particularly in the fields of proliferation, metastasis, tumor metabolism, and resistance to chemotherapy [24,25,26]. Among these, the recently unveiled lncRNA *LINC02560* has garnered significant interest because its expression patterns align with the advancement of various tumor types, pointing to its capability as an innovative biomarker and target for therapy [27,28,29]. Nonetheless, its implication in PTC remains unexplored. Within the context of this investigation, *LINC02560* was observed to exhibit heightened expression in both PTC cells and tumor tissues, demonstrating a direct association with the malignant progression and unfavorable prognosis of PTC. Notably, *LINC02560* knockdown substantially inhibited the proliferation, migration, and invasiveness of PTC cells both in vitro and in vivo. These findings strongly indicate that *LINC02560* functions as an oncogenic element in PTC and, thus, has promise as a biological indicator for predicting adverse prognoses in PTC.

The biological functions of RNAs are strongly associated with subcellular localization [30]. This study found that *LINC02560* predominantly localizes to the cytoplasm of PTC cells and binds to Ago2 protein, suggesting that it might function via the classical ceRNA network. Bioinformatics databases predicted miRNAs that could interact with *LINC02560*, while CHIRP, dual-luciferase reporter, and RIP assays identified *miR-505-5p* as having high binding affinity to *LINC02560*. In lung adenocarcinoma, *miR-505-5p* acts as an oncogene by targeting TP53AIP1 to inhibit apoptosis [31]. Conversely, in breast cancer, it acts as a tumor-suppressive miRNA by inducing apoptosis and inhibiting proliferation [32]. Our analysis demonstrated that *miR-505-5p* was considerably downregulated in PTC tissues and showed a negative correlation with *LINC02560* expression. The suppressive impact of *LINC02560* knockdown on PTC cell growth and movement was reversed by treatment with a *miR-505-5p* inhibitor, as confirmed by functional rescue experiments. Further investigation of *miR-505-5p* targets using mirMAP and TargetScan predicted PDE4C, a phosphodiesterases (PDEs) family member, as a target. PDEs are the primary enzyme family responsible for cyclic nucleotide degradation by hydrolyzing cyclic nucleotides into their inactive forms [33]. Dysregulation of this signaling is a hallmark of the disease and is associated with cancer progression [34,35,36,37]. High PDE4C expression is associated with TC progression and poor survival outcomes [35]. In lung cancer, PDE4C is associated with the tumor suppressor miR-542-5p [37]. To the best of our knowledge, this is the first study to report PDE4C as a downstream target of *miR-505-5p* in PTC, where *miR-505-5p* upregulation significantly suppresses PDE4C expression. Rescue experiments further demonstrated that co-transfection of the *miR-505-5p* inhibitor with *LINC02560* knockdown reversed PTC cell proliferation, invasion, and migration, while increasing PDE4C mRNA levels. This confirms that *LINC02560* sequesters *miR-505-5p*, reducing its inhibitory effect on PDE4C, thereby promoting PTC cell proliferation, migration, and invasion and providing a novel mechanism for post-transcriptional regulation by *LINC02560*.

Transcriptome sequencing of *LINC02560* knockdown or overexpression in PTC cells revealed involvement in various cancer-related pathways, particularly the AKT pathway. *LINC02560* overexpression significantly increases AKT phosphorylation, suggesting it as an upstream regulator of this critical signaling cascade. The AKT pathway is crucial for cell survival, growth, and metabolism in various cancers, including TC [38,39,40]. The role of *LINC02560* in activating AKT signaling underscores its potential as a therapeutic target, especially for inhibiting PTC growth and metastasis. This aligns with reports that PDE4C influences AKT signaling [41,42]. Additionally, we identified the transcription factor HNF4α as a regulator of *LINC02560* expression using the RNAInter database. HNF4α, a member of the nuclear hormone receptor superfamily, is enriched in hepatocytes and linked to tumorigenesis [43]. It inhibits hepatocyte EMT and cancer stem cell formation via the β-catenin pathway [44,45] and plays a role in lncRNA transcriptional activation [46,47]. HNF4α transcription increases the expression of the lncRNA BC200, which is involved in regulating IMA development [46]. Our findings suggest that HNF4α directly regulates *LINC02560* expression by binding to its promoter region, with HNF4α transcription enhancing *LINC02560* expression. This promotes PTC progression by sponging *miR-505-5p* to target PDE4C.

This study has several notable limitations. First, the small sample size may restrict the generalizability of our findings, necessitating a larger cohort to validate the expression and function of *LINC02560* in PTC. Additionally, the absence of a comprehensive clinical correlation limits conclusions regarding *LINC02560*’s prognostic value as a biomarker. Future studies should focus on increasing the sample size and validating *LINC02560*’s role across various clinical contexts to elucidate its biological significance in PTC.

## 5. Conclusions

Overall, this study underscores the critical role of *LINC02560* in the progression of PTC, demonstrating its function as a molecular sponge for *miR-505-5p* and its impact on cellular behavior via the PDE4C and AKT signaling pathways. Our findings pave the way for future targeted therapeutic strategies against *LINC02560*, with significant potential for clinical applications. Further investigation of *LINC02560*’s biological mechanisms may yield novel insights into early diagnosis and personalized cancer treatment.

## Data Availability

The original contributions in the study are included in the articles/Supplementary Materials. For further inquiries, please contact the corresponding author.

## Supplementary Materials

The following supporting information can be downloaded at: https://www.mdpi.com/article/10.3390/biom15050630/s1, Figure S1. Overexpression of *LINC02560* promoted the metastasis and growth of PTC cells in vitro. Figure S2. Inhibition of PTC cell proliferation, migration, and invasion by *miR-505-5P* in vitro. Figure S3. PDE4C knockdown inhibited the metastasis and growth of PTC cell in vitro. Figure S4. HNF4α knockdown inhibited the metastasis and growth of PTC cell in vitro. Figure S5. *LINC02560* partially reverses the tumor-promoting effect of HNF4α. Table S1. List of Plasmids, miRNA mimics, and miRNA inhibitor; Table S2. RT primer sequence; Table S3. The *LINC02560* ChIRP probe sequence.
